# Differential Expression of Mitogen-Activated Protein Kinase Signaling Pathways in the Human Choroid–Retinal Pigment Epithelial Complex Indicates Regional Predisposition to Disease

**DOI:** 10.3390/ijms251810105

**Published:** 2024-09-20

**Authors:** Dylan R. Hailey, Debolina Kanjilal, Peter Koulen

**Affiliations:** Vision Research Center, Department of Ophthalmology, School of Medicine, University of Missouri–Kansas City, Kansas City, MO 64108, USA

**Keywords:** MAPK signaling, retinal disease, regional protein expression, RPE

## Abstract

The retina is composed of neuronal layers that include several types of interneurons and photoreceptor cells, and separate underlying retinal pigment epithelium (RPE), Bruch’s membrane, and choroid. Different regions of the human retina include the fovea, macula, and periphery, which have unique physiological functions and anatomical features. These regions are also unique in their protein expression, and corresponding cellular and molecular responses to physiological and pathophysiological stimuli. Skeie and Mahajan analyzed regional protein expression in the human choroid–RPE complex. Mitogen-Activated Protein Kinase (MAPK) signaling pathways have been implicated in responses to stimuli such as oxidative stress and inflammation, which are critical factors in retina diseases including age-related macular degeneration. We, therefore, analyzed the Skeie and Mahajan, 2014, dataset for regional differences in the expression of MAPK-related proteins and discussed the potential implications in retinal diseases presenting with regional signs and symptoms. Regional protein expression data from the Skeie and Mahajan, 2014, study were analyzed for members of signaling networks involving MAPK and MAPK-related proteins, categorized by specific MAPK cascades, such as p38, ERK1/2, and JNK1/2, both upstream or downstream of the respective MAPK and MAPK-related proteins. We were able to identify 207 MAPK and MAPK-related proteins, 187 of which belonging to specific MAPK cascades. A total of 31 of these had been identified in the retina with two proteins, DLG2 and FLG downstream, and the other 29 upstream, of MAPK proteins. Our findings provide evidence for potential molecular substrates of retina region-specific disease manifestation and potential new targets for therapeutics development.

## 1. Introduction

The human retina consists of neurosensory layers and the separate underlying retinal pigment epithelium (RPE), Bruch’s membrane, and choroid. While the neurosensory layers of the retina, which include photoreceptor cells and interneurons, such as bipolar cells, horizontal cells, amacrine cells, and ganglion cells, are needed to process and transmit visual information [1], the RPE is involved in physiological and metabolic support of the photoreceptor cells and contributes to the optical properties of the eye [2,3]. RPE cells are known to phagocytose and degrade or recycle components of the neurosensory layers of the retina [2]. The human retina displays at least three distinct anatomical regions—the fovea, macula, and peripheral retina—with the macula providing central vision and the fovea being responsible for the highest acuity central vision. While cellular and subcellular differences in the three regions are well known for the neuronal layers, recent evidence also points towards significant regional differences for both RPE and choroid [4,5]. While retinal diseases such as age-related macular degeneration (AMD), central serous chorioretinopathy, Stargardt Disease, Doyne honeycomb retinal dystrophy, central geographic atrophy and others preferentially affect central regions of the retina, differential regional susceptibility and disease development are also typical for a wide range of other diseases such as different forms of glaucoma and diabetic retinopathy. AMD pathogenesis involves a complex range of signaling pathways and factors primarily affecting the macula and fovea [6]. In general, disease development is determined not only by the etiology of the insult, but also by the cellular and molecular responses to the insulting factors [7]. Ophthalmologic diseases are no different, with enzymatic pathways dictating the cellular response to inflammation, ischemia, hypoxia, oxidative stress, cytokines, radiation, injury, or growth factors. Therefore, a differential regional expression of unique enzymatic signaling pathways could help explain the regional susceptibility to various retinal diseases and potentially identify new therapeutic strategies. As Mitogen-Activated Protein Kinase (MAPK) pathways play a role in cellular and molecular responses to external and internal stimuli such as oxidative stress, growth factors, and inflammation [8], these pathways regulate a wide range of cellular processes such as apoptosis, cellular protection and survival, proliferation, differentiation, and motility [9]. Here, we analyzed a dataset of the human choroid–RPE complex proteome [10] for proteins of MAPK signaling pathways that are differentially expressed in distinct regions of the choroid–RPE complex and discuss potential implications for retinal disease that show regionally distinct pathogenesis and pathology.

## 2. Results

**Figure 1:**
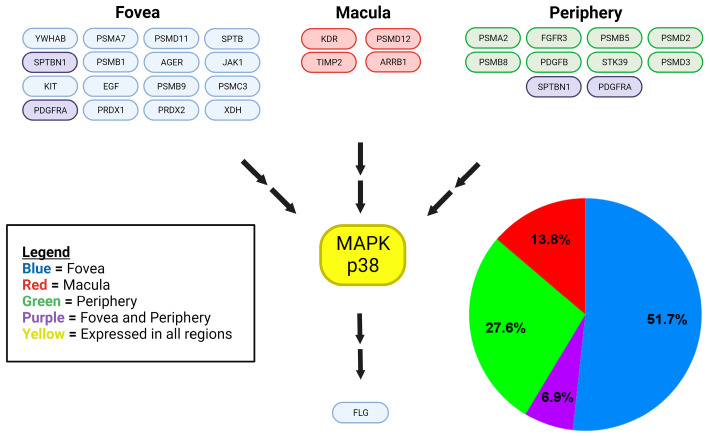
Regional expression of proteins identified within the p38 MAPK signaling cascade. MAPK-related proteins of the p38 MAPK signaling cascade are represented in the protein-signaling diagram. Blue color-coded proteins represent those detected in the foveal choroid–RPE only. Green color-coded proteins represent those detected in the peripheral choroid–RPE only. Purple color-coded proteins represent those detected in peripheral and foveal, but not the macular choroid-RPE. Red color-coded proteins represent those detected in the macular choroid–RPE only, while yellow color-coding indicates proteins detected in all regions. Double arrows represent an indirect interaction or downstream effect with any number of protein interactions in between. MAPK-related proteins of the p38 MAPK signaling cascade are also analyzed quantitatively in a pie chart illustrating the relative expression of proteins in each region.

**Figure 2:**
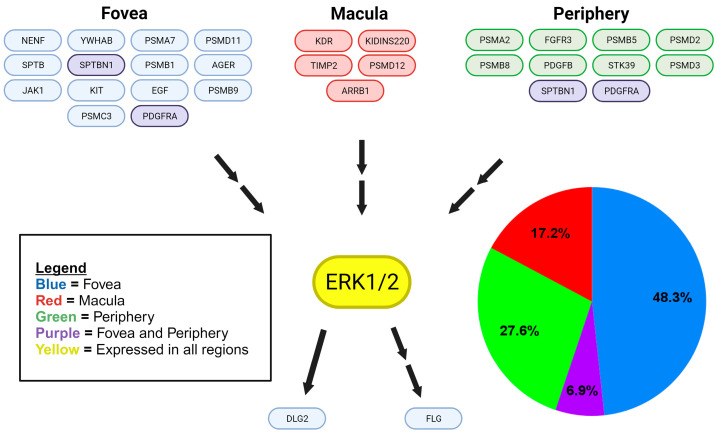
Regional expression of proteins identified within the ERK signaling cascade. MAPK-related proteins of the ERK signaling cascade are represented in the protein-signaling diagram. Blue color-coded proteins represent those detected in the foveal choroid–RPE only. Green color-coded proteins represent those detected in the peripheral choroid–RPE only. Purple color-coded proteins represent those detected in peripheral and foveal, but not the macular choroid-RPE. Red color-coded proteins represent those detected in the macular choroid–RPE only, while yellow color-coding indicates proteins detected in all regions. Single arrows represent a direct interaction, while double arrows represent an indirect interaction or downstream effect with any number of protein interactions in between. MAPK-related proteins of the ERK signaling cascade are also analyzed quantitatively in a pie chart illustrating the relative expression of proteins in each region.

**Figure 3:**
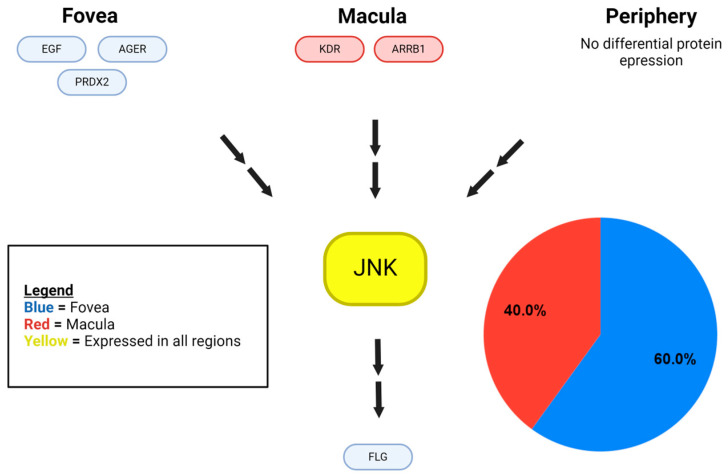
Regional expression of proteins identified within the JNK signaling cascade. MAPK-related proteins of the JNK signaling cascade are represented in the protein-signaling diagram. Blue color-coded proteins represent those detected in the foveal choroid–RPE only. Green color-coded proteins represent those detected in the peripheral choroid–RPE only, of which there are none detected in the JNK pathway. Purple color-coded proteins represent those detected in peripheral and foveal, but not the macular choroid-RPE, of which there are none detected in the JNK pathway. Red color-coded proteins represent those detected in the macular choroid–RPE only, while yellow color-coding indicates proteins detected in all regions. Double arrows represent an indirect interaction or downstream effect with any number of protein interactions in between. MAPK-related proteins of the JNK signaling cascade are also analyzed quantitatively in a pie chart illustrating the relative expression of proteins in each region.

We identified a total of 203 MAPK proteins and MAPK-related proteins in eTable 1 [10]. We categorized 187 of these proteins based on the MAPK pathway they affected. We found 31 MAPK-related proteins within the choroid–RPE of the retina, which were identified from eTable 4, and we categorized them based on location (fovea, macula, periphery) and which MAPK pathway was influenced (Figure 1, Figure 2 and Figure 3).

Our analysis revealed that differentially expressed proteins within the foveal region of the choroid–RPE included the epithelial growth factor (EGF), platelet-derived growth factor receptor alpha (PDGFRA), mast/stem cell growth factor receptor Kit (KIT), advanced glycosylation end product-specific receptor (AGER), neudesin (NENF), tyrosine-protein kinase (JAK1), disks large homolog 2 (DLG), filaggrin (FLG), 14-3-3 protein beta/alpha (YWHAB), proteasome subunit alpha type-7 (PSMA7), 26S proteasome non-ATPase regulatory subunit 11 (PSMD11), proteasome subunit beta type-1 (PSMB1), proteasome subunit beta type-9 (PSMB9), 26S proteasome regulatory subunit 6A (PSMC3), peroxiredoxin-1 (PRDX1), peroxiredoxin-2 (PRDX2), spectrin beta chain, erythroytic (SPTB), and xanthine dehydrogenase/oxidase (XDH). In the macula, differentially expressed proteins included vascular endothelial growth factor 2 (KDR/VEGFR2), arrestin beta 1 (ARRB1), metalloproteinase inhibitor 2 (TIMP2), 26S proteasome non-ATPase regulatory subunit 12 (PSMD12), and kinase D-interacting substrate of 220 kDa (KIDINS220). Lastly, differentially expressed proteins in the peripheral choroid–RPE include platelet-derived growth factor subunit B (PDGFB), platelet-derived growth factor receptor alpha (PDGFRA), fibroblast growth factor receptor 3 (FGFR3), proteasome subunit alpha type-2 (PSMA2), proteasome subunit beta type-5 (PSMB5), proteasome subunit beta type-8 (PSMB8), 26S proteasome non-ATPase regulatory subunit 2 (PSMD2), 26S proteasome non-ATPase regulatory subunit 3 (PSMD3), and STE20/SPS1-related proline-alanine-rich protein kinase (STK39). Proteins differentially expressed in both foveal and peripheral regions, but not the macular choroid–RPE, include PDGFRA and spectrin beta chain, and non-erythrocytic 1 (SPTBN1).

Of the retinal choroid–RPE proteins identified in eTable 4 that are components of the p38 MAPK signaling pathway, 51.7% were found in the fovea, 27.6% in the periphery, 13.8% in the macula, and 6.9% in both the fovea and the periphery, with p38 MAPK identified in all regions (Figure 1). Similarly, ERK1/2 was detected in all regions of the retinal choroid–RPE and ERK MAPK pathway proteins (identified in eTable 4), displaying a differential distribution with 48.3% found in the fovea, 27.6% in the periphery, 17.2% in the macula, and 6.9% in both the fovea and the periphery of the choroid–RPE(Figure 2). While JNK was also detected in all regions of the retina choroid-RPE, proteins of the JNK MAPK pathway (identified from eTable 4) were fewer in number and found only in the central retina, with 60% detected in the fovea and 40% in the macula of the choroid–RPE (Figure 3). In terms of overall differential distribution patterns, the largest number of MAPK-related proteins from the three MAPK pathways—p38, JNK, and ERK—were identified in the fovea of the choroid–RPE (18 in total; Figure 1, Figure 2 and Figure 3). In contrast, the diversity of proteins from the three MAPK pathways was significantly lower in the macular region of the choroid–RPE, with only five proteins detected; we identified 10 proteins differentially expressed in the peripheral choroid–RPE (Figure 1, Figure 2 and Figure 3). While we identified two proteins of MAPK signaling pathways expressed in both the fovea and the periphery of the choroid-RPE, proteins detected in the macula were unique to that region of the choroid–RPE (Figure 1, Figure 2 and Figure 3).

**Figure 4:**
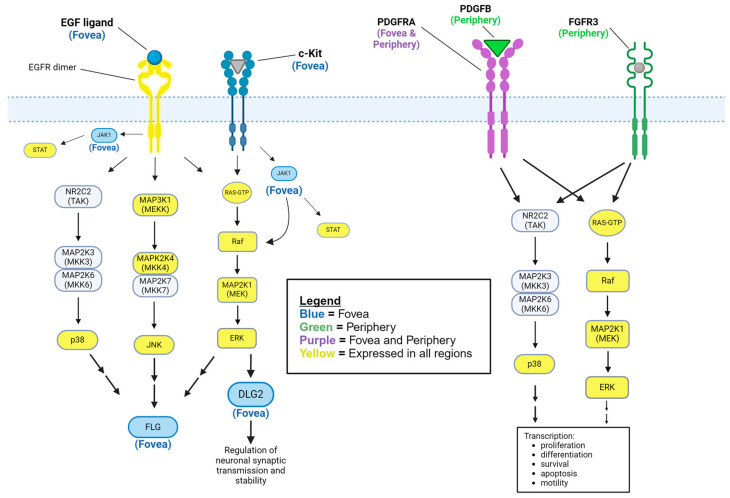
Signaling pathways in human choroid–RPE of the fovea and periphery. EGF (fovea only) binding to EGFR (all regions) activates classical p38 MAPK, ERK1/2, and JNK1/2 cascades [15,16]. KIT activates a classical ERK1/2 cascade. Both receptors activate JAK1 (fovea only), which activates various STAT proteins (all regions) or RAF1 of the ERK1/2 cascade [17,18]. DLG2 is activated directly by ERK1/2 and is involved in neuronal synaptic stability and transmission by regulating various channel proteins [19,20,21,22]. P38 MAPK, ERK1/2, and JNK all indirectly activate FLG (filaggrin), which is implicated in keratinization and atopic dermatitis [14,23]. PDGFB (periphery only) binding to PDGFRA (fovea and periphery) leads to activation of ERK1/2 and p38 MAPK signaling cascades [24,25]. FGFR3 (periphery only) also activates ERK1/2 and p38 MAPK signaling cascades, leading to the transcription of factors for cellular proliferation, differentiation, survival, apoptosis, and motility [26]. Blue color-coded proteins represent those detected in the foveal choroid–RPE only. Green color-coded proteins represent those detected in the peripheral choroid–RPE only. Purple color-coded proteins represent those detected in the peripheral and foveal regions, but not the macular choroid-RPE. Yellow color-coded proteins represent those detected in all regions of the choroid-RPE. Grey color-coded proteins represent those in classical signaling pathways that were not detected in any regions but are included for reference. Single arrows represent a direct interaction, while double arrows represent an indirect interaction or downstream effect with any number of protein interactions in between.

**Figure 5:**
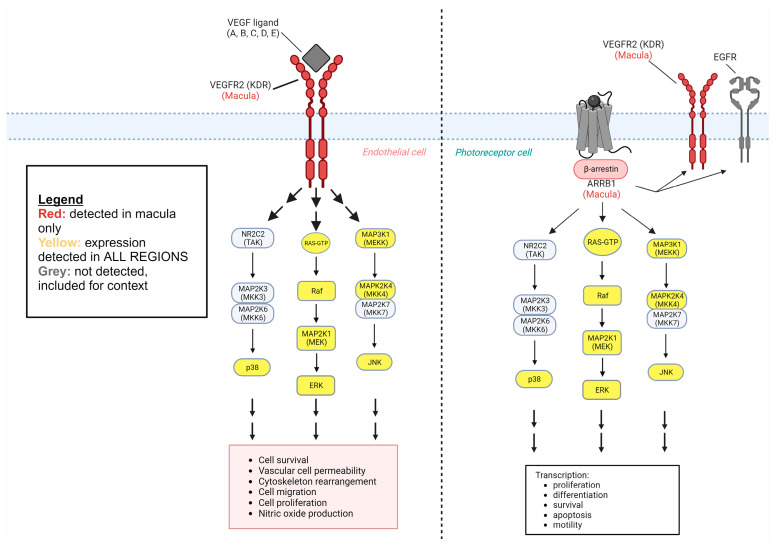
Signaling pathways in human choroid–RPE of the macula. Illustrated is a ligand representing various VEGFs binding to KDR/VEGFR2 (macula only) on the surface of an endothelial cell to activate ERK1/2 and p38 MAPK cascades subsequently [27,28]. These cascades lead to the transcription of factors important for angiogenesis, cell migration and proliferation, and more [27,28]. ARRB1 (macula only), i.e., arrestin beta 1, is associated with a photoreceptor cell surface rhodopsin GPCR and has not only been shown to activate ERK1/2 and p38 MAPK cascades, but also transactivate nearby membrane receptors like KDR/VEGFR2 and EGFR, and their subsequent cascades without the need for the binding of their respective ligands [29,30,31,32,33]. It is important to remember that this is a proteomic study of the choroid–RPE complex, not neurosensory layers of the retina where ARRB1 is known to be expressed. Red color-coded proteins represent those detected in the macular choroid–RPE complex only. Yellow color-coded proteins represent those detected in all regions of the choroid–RPE complex. Grey color-coded proteins represent those in classical signaling pathways that were not detected in any regions but are included for reference. Single arrows represent a direct interaction, while double arrows represent indirect interaction or downstream effect with any number of proteins in between.

While most identified proteins were upstream components of the MAPK signaling pathways (Figure 1, Figure 2, Figure 3, Figure 4 and Figure 5), we also determined from eTable 4 established signaling proteins downstream to MAPK and they also displayed significant differential distribution. Only in the fovea were two choroid–RPE proteins detected that were controlled by MAPK signaling. DLG2 has been described as directly and indirectly activated by ERK1/2 [11,12] (Figure 4). FLG has been characterized as indirectly regulated by MAPK proteins, and has been found to be upregulated by ERK and p38 MAPK [13], and ERK, JNK, and p38 MAPK [14].

## 3. Discussion

A total of 31 of the 187 MAPK-related proteins were identified as having a differential regional expression within the human choroid–RPE complex, with the remaining MAPK-related proteins were expressed in all regions. This preserved expression in all regions indicates a likely overlap in the stimuli to which the cells of each region respond, and how they respond to these stimuli. The 31 regionally expressed MAPK-related proteins indicate that these regions also differ in the stimuli to which they respond and the manner in which they respond. There was unique regional protein expression in various cascades of each of the three MAPK proteins considered in this study: p38 MAPK, ERK1/2, and JNK 1/2 (Figure 1, Figure 2 and Figure 3). Region-specific responses to various stimuli may contribute to the regional presentation of retinal diseases. Consequently, the inspection of specific differentially expressed proteins and their functions enables the identification of potential molecular substrates for pathologic mechanisms of diseases affecting specific regions of the retina.

### 3.1. Regionally Presenting Retinal Disease

There are two major forms of AMD: non-exudative and exudative AMD. Non-exudative or “dry” AMD is characterized by lipofuscin deposits or drusen in the macula, associated with early atrophy of the RPE and with geographic atrophy (GA) as an advanced, late-stage form of non-exudative AMD [34]. Both dry AMD and GA are characterized by slowly progressive vision loss secondary to drusen and atrophy of the outer retinal layers, respectively [34]. With onset often in the setting of pre-existing dry AMD, neovascular AMD is associated with the initial impairment of choroidal vasculature leading to rapid choroidal neovascularization, where vision loss can occur rapidly due to subretinal fluid accumulation, intraretinal and preretinal hemorrhage, vitreous hemorrhage, and retinal detachment [34,35].

On a molecular and cellular level, AMD is caused by a combination of factors such as metabolic differences, oxidative stress, inflammation, and extracellular matrix (ECM) structural differences [36,37,38,39]. Separately, angiogenic factors are related to the pathogenesis of neovascular AMD [40]. MAPK pathways are known to mediate many of these cellular processes. Of the MAPK-related proteins we were able to identify in the Skeie and Mahajan, 2014 datasets, with several potentially being implicated in AMD pathogenesis. Coding for VEGFR2, KDR was detected in the macula and absent in the fovea and periphery. Vascular endothelial growth factors (VEGFs) bind VEGFR2 to promote angiogenesis and endothelial cell migration. KDR has not been directly linked to the development of neovascular or exudative AMD [41,42]. Though a causal relationship to pathogenesis has yet to be identified, this protein likely still contributes to neovascularization given its general cellular function and its expression in the macula. One 2018 study found that RPE exosomes released greater amounts of VEGF2 when under oxidative stress, but when VEGFs were inhibited, angiogenesis still occurred, indicating another pathway leading to neovascularization must be present in AMD [43]. ARRB1, also expressed exclusively in the macula, has been implicated in the transactivation of VEGFR2/KDR without the need for extracellular ligand binding, as depicted in Figure 2 [32]. This could explain persistent angiogenesis despite the inhibition of VEGF binding to receptors, though ARRB1 is currently known to be expressed only by photoreceptor cells in the neurosensory layers of the retina [33]. Arrestins bind intracellular domains of GPCRs and modulate their downstream signaling pathways, thus implicating themselves in a broad range of cellular functions. ARRB1 or arrestin beta 1, is involved in the inhibition of visual signaling by binding rhodopsin GPCR in photoreceptor cells of the retina [33]. This location of ARRB1 in cells of neurosensory layers is notable because the Skeie and Mahajan study analyzed protein expression in a tissue sample of choroid–RPE complexes only, and if endogenous expression can be ruled out, the phagocytosis of photoreceptor cells’ outer segments by RPE cells could cause the positive identification of proteins traditionally recognized as photoreceptor cell-specific. ARRB1 has also been found to regulate cellular immunity through the TLR-IL1R signaling pathway with ARRB1-deficient mice showing increased release of pro-inflammatory cytokines [44]. Similarly, TIMP2 is expressed in the macula but not in the fovea or the periphery. TIMP2 is involved in modulating metalloproteinase activity and response to oxidative stress in the RPE [45], but likely not associated with AMD [46]. On the other hand, TIMP3 has been associated with AMD [47], which was found in all regions of the choroid–RPE in the study by Skeie and Mahajan, 2014. 

On the other hand, expressed in both the periphery and the fovea but not the macula is PDGFRA, which is implicated in VEGF signaling, fibroblast proliferation, and the cellular response to reactive oxygen species (ROS) [48,49,50]. Figure 3 depicts MAPK signaling pathways downstream of PDGFRA. These are cellular functions that are involved in the development of AMD, and the protein’s absence from the macula of the healthy eyes studied by Skeie and Mahajan, 2014, is likely due to the absence of AMD. This could be tested in future studies by analyzing and comparing macular PDGFRA expression of both healthy and AMD-affected eyes. Another protein absent from the macula but present elsewhere in the retina is SPTBN1. SPTBN1 has been implicated in neuronal cytoskeletal structure and variants have been associated with various aberrancies during neurological development [51]. It is unclear if this could be associated with the development of AMD, but a lack of SPTBN1 expression in the macula indicates a potential vulnerability that might play a role in AMD development.

There is also noticeable variability in the expression of particular proteasome components with the macular choroid–RPE showing expression of only PSMD12, the foveal choroid–RPE showing expression of PSMA7, PSMD11, PSMB1, PSMB9, and PSMC3, and the peripheral choroid–RPE showing expression of PSMA2, PSMB5, PSMB8, PSMD2, and PSMD3. These proteins are all components of the 26s proteasome complex involved in the degradation or reactivation of misfolded or otherwise ubiquitinated proteins, preventing their accumulation [52,53]. The RPE is largely responsible for the phagocytosis of photoreceptor cell components [2]. Differences in proteasome complex expression in the macula could help explain its susceptibility to AMD pathogenesis due to deficiencies in the degradation of misfolded proteins and the regulation of protein concentration, including but not limited to lipofuscin accumulation, or drusen. Variants of PSMD12 or deficiencies in PSMD12 expression in the macula could also potentially contribute to the region-specific phenotype of AMD development. 

### 3.2. Downstream Proteins

Based on the proteins that were detected and determined to be downstream of MAPK pathways FLG and DLG2, we can correlate their general function to potential disease implications within the choroid–RPE complex. FLG codes for the protein filaggrin, which gene ontology analysis revealed is broadly implicated in keratinization and as an extracellular structural component [14,23]. Notably, mutations in this gene are known to cause atopic dermatitis and ichthyosis vulgaris [54,55]. While these are primarily dermatologic conditions, atopic dermatitis has known ophthalmologic complications, such as blepharitis, conjunctivitis, and, most relevantly, retinal detachment [56]. While it is possible that FLG mutations could be implicated in this increased rate of retinal detachment, this idea is made less likely by the fact that retinal detachments in atopic dermatitis have tended to occur in the periphery and are theorized to be secondary to mechanical disturbance of the eye with rubbing or itching [57]. In our study, we found FLG to be differentially expressed at the fovea as opposed to the periphery. There are no studies that specifically correlate FLG or COL7A1 dysfunction with retinal detachment. However, the role of FLG in the RPE-choroid complex appears deserving of further study.

DLG2 is thought to be expressed in neuronal cell membranes at synapses, regulating their stability and signal transduction [19]. It is known to interact with, and regulate, the NMDA receptor, AMPA receptor, and potassium channel expression [20,21,22], as depicted in Figure 4. This result has to be considered in the context of the scope of the Skeie and Mahajan study, which targeted the choroid-RPE and served as the data source of the present study. Deletions have been associated with schizophrenia, which has been associated with retinal structural and functional changes [58,59]. While there is no clear predilection for these changes in retinal structure and function in particular regions of the retina of patients diagnosed with schizophrenia, it is possible that DLG2, while expressed exclusively in the fovea, contributes to these retinal changes. DLG2 is activated by FYN [60] and is known to interact with DLGAP1 and DLG1, a paralog of DLG2 [61]. Both DLGAP1 and DLG1 are associated with retinitis pigmentosa 55, a disorder characterized by photoreceptor loss and reactive pigment changes in the RPE starting in the mid-peripheral fundus [62,63,64]. Again, in our study, DLG2 was found to be differentially expressed in the fovea, making involvement in the pathophysiology of retinitis pigmentosa less likely.

*Limitations*: In the Skeie and Mahajan, 2014 proteome dataset, there were more proteins identified upstream of MAPK proteins than there were downstream. There are various potential interpretations for this finding. We could conclude from this that there is simply far less expression of proteins that MAPK proteins either up-regulate or down-regulate. We can also consider the ability of the tissue sampling, processing, and subsequent protein identification processes to detect low-abundance proteins [65]. This, in combination with the relatively small proteome dataset in the Skeie and Mahajan, 2014 study, represents our study’s most significant limitations. For future studies, we recommend using larger datasets of detected proteins from tissue samples. Another limitation of this study is that the tissue samples were taken from only three healthy retinas [10]. A greater number of tissue samples could improve the number of detected proteins expressed in particular regions of the retina and provide a more exhaustive list. Future studies could compare the protein expression of healthy retinas to that of diseased retinas of a particular pathology to determine if there are any differentially expressed MAPK-related proteins that could help explain disease pathophysiology. A proteomic study, like that of Skeie and Mahajan, measures the presence of proteins in a tissue sample, while a genomics study measures genetic expression. In a proteomic study, proteins not expressed by the cells of a tissue sample can be present and detected due to their presence in blood, given the vascular nature of the choroid. Another mechanism by which proteins not expressed by resident cells of the choroid–RPE sample can be detected in a proteomic study is through cellular uptake or phagocytosis. This is possible in the Skeie and Mahajan study, considering the function of RPE cells as they phagocytose and recycle photoreceptors and components of the adjacent neurosensory layers. In both proteomic studies and gene analysis studies, proteins not explicitly expressed by the choroid–RPE cells sampled can be detected in cases of inadequate surgical microdissection. In the study by Skeie and Mahajan, to test tissue samples of human choroid–RPE only, samples had to be collected and prepared by dissecting the neurosensory layers of the retina. This process has the potential to leave behind unwanted cells. This issue is highlighted by the detection of a particular protein, ARRB1, which was found exclusively in the macula. This protein is known to be expressed in rod photoreceptor cells [33], and thus could have been detected in the tissue samples of the Skeie and Mahajan study by this mechanism of phagocytic uptake by RPE cells or by inadequate microdissection. In this way, the nature of a proteomic study represents a potential limitation.

## 4. Materials and Methods

Skeie and Mahajan detected 4204 proteins in the periphery, 4595 proteins in the macula, and 4409 proteins in the fovea for a total of 5438 unique proteins in the choroid–RPE samples taken from healthy eyes of three individuals aged 80+ using mass spectrometry [10]. These data were utilized to create the initial framework of proteins to analyze for our study. We utilized protein expression data of the human choroid–RPE (eTables 1 and 4) of the Skeie and Mahajan study for the present study. ETable 1 contained all the proteins identified in the human choroid–RPE by their Ensembl ID, gene ID, description, the number of unique peptides, the number of peptide hits, and the average peptide hits for each sample type. ETable 2 contained the differentially expressed proteins in the fovea, macula, and periphery of the human choroid-RPE. We expanded eTable 1 using the Ensembl IDs to retrieve corresponding UniProt (https://www.uniprot.org/ accessed on 9 October 2023) entries. We were able to map 4717 out of the 5438 Ensembl Protein identifiers from eTable 1, while 721 identifiers were unable to be mapped with UniProt. The expanded table included information on protein name (Ensembl ID), gene names, entry, entry name, protein names, gene ontology (biological process), gene ontology (cellular component), gene ontology (molecular function), gene ontology (GO), intramembrane, subcellular location [CC], topological domain, transmembrane, interacts with, subunit structure [CC], and cross-reference (reactome). Thus, we expanded eTable 1 utilizing UniProt data from which MAPK and MAPK-related proteins [7] of the human choroid–RPE were identified. Specifically, a total of 203 MAPK and MAPK-related proteins were found, of which 187 could be classified as upstream or downstream proteins in MAPK signaling cascades (p38, ERK1, ERK2, JNK1, JNK2). Classification into the respective MAPK signaling cascades was based on a thorough literature review of UniProt entries, GeneCards (https://www.genecards.org/ accessed on 9 October 2023) database, and published papers that correlated proteins to certain MAPK cascades with direct experimental evidence, such as validation by molecular biology techniques and/or protein binding studies. This list of proteins was then cross-referenced with eTable 4 to identify those MAPK-related proteins with differential regional expression within the human choroid–RPE complex. Results were summarized in diagrams that specified the MAPK-related proteins, location in the retina, and affected MAPK pathways (Figure 1, Figure 2 and Figure 3). For each MAPK pathway, the associated genes of the relevant proteins were categorized by location in the choroid–RPE of the retina (fovea, macula, and periphery), and a pie chart was generated to illustrate the relative expression of genes in each region, and then included in Figure 1, Figure 2 and Figure 3. 

Having determined proteins involved in MAPK pathways and their regional expression, we then determined downstream targets controlled by ERK1/2, JNK1/2, and/or p38 within the human choroid–RPE proteome and components of the MAPK pathways that were not detected, but that can be postulated to be present based on previous mechanistic studies.

When referring to fovea, macula, and periphery in the present study, we refer to the choroid–RPE complex of the fovea, macula, and periphery, respectively, as the datasets were derived from choroid–RPE samples [10].

## Data Availability

The original contributions presented in the study are included in the article, further inquiries can be directed to the corresponding author.
